# Relationships of Lower Lung Fibrosis, Pleural Disease, and Lung Mass with Occupational, Household, Neighborhood, and Slate Roof-Dense Area Residential Asbestos Exposure

**DOI:** 10.3390/ijerph15081638

**Published:** 2018-08-02

**Authors:** Dongmug Kang, Yu-Young Kim, Minseung Shin, Min-Su Lee, Hee-Joo Bae, Se-Yeong Kim, Young-Ki Kim

**Affiliations:** 1 Preventive, Occupational and Environmental Medicine, School of Medicine, Pusan National University, Yangsan 50612, Korea; kangdm@pusan.ac.kr; 2 Occupational and Environmental Medicine, Pusan National University Yangsan Hospital, Yangsan 50612, Korea; 30white@hanmail.net; 3 Premedical College, Pusan National University, Yangsan 50612, Korea; yyofficial96@naver.com (Y.-Y.K.); tls0264@naver.com (M.S.); leeguduk45@naver.com (M.-S.L.); heejoo700@hanmail.net (H.-J.B.); 4 Environmental Health Center for Asbestos, Pusan National University Yangsan Hospital, Yangsan 50612, Korea

**Keywords:** asbestos, domestic, environmental, health impact survey, household, Korea, neighbor, roof, slate

## Abstract

This study aimed to evaluate the relationship between various asbestos exposure routes and asbestos-related disorders (ARDs). The study population comprised 11,186 residents of a metropolitan city who lived near asbestos factories, shipyards, or in slate roof-dense areas. ARDs were determined from chest X-rays indicating lower lung fibrosis (LFF), pleural disease (PD), and lung masses (LMs). Of the subjects, 11.2%, 10.4%, 67.2% and 8.3% were exposed to asbestos via occupational, household, neighborhood, and slate roof routes, respectively. The odds ratio (OR) of PD from household exposure (i.e., living with asbestos-producing workers) was 1.9 (95% confidence interval: 0.9–4.2), and those of LLF and PD from neighborhood exposure, or residing near asbestos factories) for <19 or >20 years, or near a mine, were 4.1 (2.8–5.8) and 4.8 (3.4–6.7), 8.3 (5.5–12.3) and 8.0 (5.5–11.6), and 4.8 (2.7–8.5) and 9.0 (5.6–14.4), respectively. The ORs of LLF, PD, and LM among those residing in slate-dense areas were 5.5 (3.3–9.0), 8.8 (5.6–13.8), and 20.5 (10.4–40.4), respectively. Substantial proportions of citizens residing in industrialized cities have potentially been exposed to asbestos, and various exposure routes are associated with the development of ARDs. Given the limitations of this study, including potential confounders such as socioeconomic status, further research is needed.

## 1. Introduction

Asbestos, which is composed of long, thin fibers, has been used widely for decades because of its properties such as resistance to fire, heat, and electricity; tensile strength; and sound absorption. However, asbestos exposure can cause asbestos-related diseases (ARDs) such as malignant cancers, including lung cancer (LC), malignant mesothelioma (MM), laryngeal cancer, and ovarian cancer, as well as benign diseases such as asbestosis, pleural plaques, and pleural thickening [1]. Asbestos exposure via occupational, household, and neighborhood routes was reported as early as 1960 [2,3,4]. Occupational asbestos exposure occurs through the production of asbestos-containing products in settings such as mines and asbestos textile factories, or in workplaces that handle asbestos products, including shipyards and construction sites. Household exposure occurs when families exposed to asbestos at work come to their homes with work clothes. Neighborhood exposure occurs when living near to asbestos mines, asbestos factories, and shipyards. Although many studies have explored the extent of occupational exposure, including the associated disease burden [5,6], studies of household and neighborhood exposure to asbestos among ordinary citizens are rare. The majority of these studies indicate that ARDs consequent to household and neighborhood asbestos exposure are confined to MM [1,7]. However, most such studies did not control for other exposure routes, despite the possibility of overlap [1,2,7].

One exposure route that may be encountered during ordinary life is the asbestos slate roof, which is composed of 10–15% asbestos [8]. Asbestos slate has been widely used in western societies [9], and its use is increasing in developing countries [10]. In Korea, slate was widely used to replace roofing materials in rural areas during the period of rapid economic growth during the 1970s and 1980s, and was commonly used in poor urban areas [8]. However, few studies have addressed the health effects of living under or near slate roofs [8], even though the surrounding asbestos concentration in ambient air might be substantial [11].

In 2009, the Korean government banned asbestos. In 2011, the government began to compensate citizens for environmental asbestos diseases, and environmental health centers for asbestos (EHCA) began to conduct health impact surveys (HISs) of areas containing asbestos mines, factories, ship-building facilities, and large numbers of slate roofs [12,13]. Although HISs are based on the voluntary participation of citizens living near former sources of asbestos exposure, the responses elucidate the frequency of exposure and risks of occupational, household, and slate exposure among ordinary citizens.

The present study investigated the frequency of asbestos exposure via the occupational, household, neighborhood, and slate roof routes, and studied the relationships between these exposure routes and ARDs using HIS data of EHCA adjusted for other routes.

## 2. Materials and Methods

### 2.1. Study Subjects

The study subjects comprised participants in a HIS conducted by Pusan National University Yangsan Hospital (PNUYH) EHCA between 2009 and 2016. The majority of the participants currently reside in the Busan metropolitan area, which serves as the center of one of the most industrialized areas of Korea and traditionally housed many environmental asbestos exposure sources, such as asbestos textile factories and shipbuilding and ship repair industries [13]. For this study, the subjects were restricted to current citizens of Busan. Participants younger than 20 years were excluded to account for the long latency period of ARDs (median: 20 years) [14]. Accordingly, 2053 of the 13,433 PNUYH-EHCA HIS participants were excluded based on these criteria. After excluding an additional 194 cases because of incomplete questionnaire responses or chest radiography results, the final study comprised 11,186 subjects. The institutional review board of PNUYH approved this study (02-2009-037, 02-2012-018).

### 2.2. HIS Items: Questionnaire and Chest Radiograph

A structured questionnaire survey was administered by trained personnel through individual interviews during which informed consent was obtained from the subjects. Asbestos exposure was assessed qualitatively by the questionnaire. To determine occupational and household exposure, the subjects were asked whether they or someone in their household was exposed to structured asbestos-related work items during employment. To determine neighborhood exposure, the subject’s life residential history was taken and verified using official residential records. Additionally, the subject’s history of living under a slate roof was taken and compared to slate-dense areas predetermined by a previous study [13]. No exposure was defined as no exposure to occupational, household, or neighborhood sources, living under a slate roof, or living in slate-dense areas. 

Posteroanterior chest radiography was performed using an FCR 5501 digital radiograph device (Fuji, Tokyo, Japan). Radiographs were read by two separate radiologists who had participated in a special reading session for ARDs provided by a Korean environmental corporation. The reading results were divided into three groups of ARDs: of asbestosis, pleural disease (PD), and LC. The reading results were categorized as follows: lower lung fibrosis (LLF) (asbestosis surrogate): asbestosis, diffuse lung disease, interstitial lung disease, nodular ground glass appearance; PD: pleural plaque, pleural thickening, loss of costo-phrenic angle; and LC: suspected malignancy, mass >1 cm, mediastinal enlargement. Any case of PD with a tuberculous history or reading opinion suggesting tuberculosis was excluded. Only screening results were analyzed in this study because the participation rates in secondary examinations to address abnormal screening results were low (4.6%) and not representative [14]. 

### 2.3. Data Analysis

The X^2^-test and Fisher’s exact test were used to conduct frequency analyses, and trends were assessed using the Cochran–Armitage trend test. Classes containing small numbers were merged into adjacent classes. Logistic regressions were conducted using the three radiography outcomes (LLF, PD, and LM) as the dependent variable. Simple, multiple (adjusted for sex, age, and smoking as confounders) [3,15], and full model logistic regressions (adjusted for other exposure routes and confounders) were conducted. The statistical analysis was performed using SAS, version 9.4 (SAS Institute, Cary, NC, USA).

## 3. Results

### 3.1. Prevalence Rates of LLF, PD, and LM by Subject Characteristics and Exposre

In our study, 120 (1.9%) and 211 (3.4%) cases of LLF and PD, respectively, were observed among women, with 264 (5.3%) and 295 (5.9%) cases, respectively, among men (*p*-values for sex-based comparisons < 0.0001). Furthermore, 61 (1.0%) and 45 (0.9%) cases of LM were observed among women and men, respectively (*p*-value for comparison = 0.6063). Most patients were between 50 and 79 years of age, and the proportions of LLF, PD, and LM differed by age (*p*-values for comparisons <0.0001). Compared to present and never smokers, ex-smokers were more likely to present with LLF and PD (*p*-value < 0.0001). Of the single exposure routes, neighborhood exposure was most frequent (53.3%), followed by slate roof (5.4%), occupational (3.4%), and household (2.4%) exposure. The total proportions of neighborhood, slate roof, occupational, and household exposure were 67.2%, 8.3%, 11.2%, and 10.4% respectively. Among subjects exposed to slate roofs, 2.7% lived in slate-roof-dense areas (data not shown). 

The prevalence of LLF was highest among subjects exposed to all four sources (16.7%) followed by those exposed to occupational + neighbor + slate (13.0%) and occupational + neighbor (12.5%). Similarly, the prevalence of PD was highest among subjects exposed to all four sources (27.8%), followed by occupational + household + slate (25.0%). The prevalence of LM was highest among those exposed to slate roofs (4.2%), followed by occupational + slate (3.9%) (Table 1).

### 3.2. Occupational and Household Exposures and Radiologic Abnormalities

A total of 1257 (11.2%) subjects were occupationally exposed to asbestos (data not shown). Among them, 104 (8.3%), 95 (7.6%), and 13 (1.0%) developed LLF, PD, and LM, respectively. The highest prevalence of LLF was observed among asbestos miners (21.4%), asbestos cement manufacturers (14.3%), and asbestos textile workers (11.9%) (*p* < 0.0001). The highest prevalence of PD was also observed among asbestos miners (17.9%), followed by asbestos friction material and joint sheet manufacturers (17.2%), and asbestos textile workers (11.9%) (*p* < 0.0001). By contrast, few subjects with occupational exposure developed LM, and no differences among occupations were observed (*p* = 0.3889). Differences in the prevalence of LLF and PD were also observed according to the duration of work (*p* < 0.0001 and *p* < 0.0001, respectively).

A total of 1159 (10.4%) subjects were exposed to asbestos by other household members with asbestos-related occupations (data not shown), and 31 (2.7%), 42 (3.6%), and 4 (0.3%) developed LLF, PD, and LM, respectively. Among the occupations of household members, the highest prevalence of LLF was associated with asbestos textile work (14.2%), followed by asbestos cement production (4.5%), and the highest prevalence of PD was associated with asbestos textile work (23.8%), and asbestos friction material and joint sheet manufacturing (8.3%) (Table 2).

### 3.3. Neighborhood and Slate Roof Exposure and Slate Root-Dense Areas

A total of 7515 (67.2%) and 220 (2.0%) cases of neighborhood exposure from local asbestos factories and mines, respectively, were reported (data not shown). The prevalence of LLF, PD, and LM consequent to exposure to mines and factories were 302 (4.0%), 390 (5.2%), and 68 cases (0.9%) and 23 (10.5%), 42 (19.1%), and 3 cases (1.4%), respectively (*p*-value < 0.0001, *p*-value < 0.0001, and *p*-value = 0.6994, respectively).

Overall, 929 (8.3%) and 299 (2.7%) cases of slate roof exposure and residence in slate roof-dense areas were reported, respectively (data not shown). The prevalence of LLF, PD, and LM associated with slate roof exposure and residence in a slate roof-dense area were 35 (3.8%), 75 (8.1%), and 8 (0.9%); 390 (5.2%), and 68 (0.9%); and 31 (10.4%), 42 (14.0%), and 30 (10.0%), respectively (*p*-value < 0.0001). Differences in radiological abnormalities were observed with regard to the durations of neighborhood and slate roof exposure (*p*-value < 0.0001) (Table 3).

### 3.4. Trends in Combined Variables of Exposure Routes and Durations

Occupational, household, neighborhood, and slate roof exposures were classified as use vs. production, use vs. production, factory vs. mine, and slate roof only vs. slate roof in slate roof-dense area, respectively. Subsequently, the two categories for occupational, neighbor, and slate roof exposure were combined with durations of <20 vs. >20 years; the cut-off for household exposure duration was 5 years because many subjects did not reside with household members for as long as 20 years. The increasing trends in LLF differed among the categories for all exposure routes except household exposure (*p*-value < 0.0001). The trends in PD differed among the categories in all exposure routes and increased for all routes except household exposure. The trends in LM differed by categories for all exposure routes except household exposure, and increased with slate roof exposure (Table 4).

### 3.5. Logistic Regression Analysis

Given the small numbers of subjects in some categories, such as occupational exposure via production >20 years, household exposure via production <4 years, neighborhood mine exposure >20 years, and slate roof exposure in a dense area <19 years, these categories were merged with adjacent categories in the logistic regression analysis. The odds ratios (OR) of LLF following occupational exposure were increased in the categories of asbestos use >20 years and asbestos production. The ORs of LLF following neighborhood exposure were increased in all categories and in all models. However, for slate roof exposure, the OR of LLF was increased only in slate roof-dense areas.

The ORs of PD related to occupational exposure were similar to the ORs of LLF. The ORs were increased in the categories of asbestos use >20 years and asbestos production. However, the OR of PD following household exposure was increased only in the category of production. The ORs for PD following neighborhood exposure and slate roof exposure were increased in all categories and in all models. Regarding LM, the OR following neighborhood exposure was increased in the category of factory exposure >20 years in all models, and factory exposure <19 years only in the full model. The ORs of LLF, PD, and LM in two categories, neighborhood factory exposure >20 years and slate roof exposure in a slate roof-dense area, were increased in all models (Table 5).

## 4. Discussion

In the present study, 11.2%, 10.4%, and 8.3% of subjects were exposed to asbestos through slate roof, occupational, and household routes, respectively. We note that these exposures were identified incidentally, as the subjects participated in a HIS focused on neighborhood exposure and slate roof-dense areas. As noted, despite many previous studies of ARDs and various asbestos exposure routes [1,3,4], studies on the extent of exposure among ordinary residents are rare. The proportions in this study might have been over-estimated because individuals who were well aware of asbestos, such as occupationally exposed workers and their families, may have participated at high rates. As the proportions of various non-occupational exposure routes have not been routinely studied [6,16], the authors could not compare the frequencies of exposure via various routes with those of other studies.

Occupational exposure in the asbestos mining and asbestos textile industries was associated with higher prevalence of LLF and PD. In general, occupational exposure during the production of asbestos-containing materials was associated with higher prevalence of LLF and PD, compared to the occupational use of asbestos products [17]. A previous finding that low levels of occupational, household, and neighborhood exposure may induce pleural plaques [18] is consistent with our results. In this study, household exposure correlated only with PD. Previous studies of household exposure have reported similar results, indicating a significant relationship between household exposure and pleural abnormalities not related to lung cancer [19]. Although many studies identified pleural abnormalities related to household exposure without controlling for confounders [19], the present study adjusted for both confounders and other exposure routes.

The prevalence levels of PD were similar among those who resided near mines for <19 years and near factories for >20 years. Although the ambient concentrations of asbestos fibers may differ according to the source [13], previous studies have not addressed the effects of various exposure sources, including mines, factories, and ship-building facilities, on environmental exposure. Furthermore, the prevalence of PD increased among people who resided in slate roof-dense areas. Although asbestosis normally occurs at relatively high exposure levels, with the exception of mild fibrosis occasionally occurring at lower levels [18], the high prevalence of PD resulting from neighborhood exposure and slate roof exposure in a slate-dense area was unusual. The U.S. Occupational Safety and Health Administration has set a permissible exposure limit for workers of 0.1 asbestos fibers/cc in a time weighted average of 8 h [20]. However, a reference dose for non-malignant ARDs is not available, and it is difficult to determine the concentration or exposure level required to induce asbestosis.

Slate roofs can be damaged by age, rain, and wind [21]. Although a previous ecological study predicted future mortality due to asbestos slate roofs, particularly due to malignant mesothelioma [8], the study focused on asbestos consumption in general, and not on slate roof exposure. Another study found that some of the indoor air concentrations of asbestos in slate roof houses in Busan city exceeded 1 × 10^−4^ f/mL [22]; although this might not support a high incidence of PD in in slate roof-dense areas, it might support the development of LC. According to the US Environmental Protection Agency, specific risk levels of combined LC and MM that cause extra cancer in relation to air concentrations are 1 in 10,000 for 4 × 10^−4^ f/mL, 1 in 100,000 for 4 × 10^−5^ f/mL, and 1 in 1,000,000 for 4 × 10^−6^ f/mL [23].

In short, this study indicated an increased cancer risk of >1 in 10,000 among residents with slate roofs. Therefore, living under a slate roof might lead to ARDs. However, given the low sensitivity and specificity of chest X-rays, the radiologic results in the present study might not have detected genuine ARDs. People who live under slate roofs and/or in slate-dense areas tend to have a lower socioeconomic status (SES), which is associated with other lung diseases such as idiopathic pulmonary fibrosis [24,25]. Accordingly, the relationship between LM and a slate-dense area in the present study may be attributable to a lower SES [26]. We note that ours may have been the only study to investigate ARDs consequent to living under a slate roof and/or in a slate-roof-dense area.

This study had some limitations of note. First, both exposures and outcomes may have been misclassified. Occupational, household, and slate roof exposure data relied on the memories of the research subjects. However, as information about residential histories and slate roof-dense areas was verified using official records, this bias might not apply to neighborhood exposure and slate-roof-dense areas. Additionally, this study was based on the results of screening tests such as chest radiography, rather than on specific confirmatory tests such as computed tomography and clinical diagnosis. This may have led to the over- or under-estimation of disease prevalence. To avoid the misclassification of tuberculosis sequelae such as pleural plaques or costo-phrenic angle blunting, we excluded cases with histories of tuberculosis from the PD category. Despite these efforts to prevent misclassification, the inherent limitations of the HIS dataset may have introduced substantial bias. Second, a subject with respiratory symptoms might have been more likely to recall exposure sources, leading to information bias. Third, although age, sex, and smoking status were controlled as confounders, SES was not investigated, despite its role as a potential confounder. This variable should be controlled in future studies, as subjects exposed to asbestos may have had a lower SES. Fourth, the study subjects were voluntary participants, which reduces the generalizability of the results. Finally, exposure should require the evaluation of the fiber number in the living area and/or the patient sputum, our study is speculative and presently only indicative. It should be subsequently confirmed by the objective evaluation of the exposure. Also, Korea used almost chrysotile asbestos, so the results cannot be generalized to other situations in which amphiboles were mainly used.

These limitations warrant further studies that include objective exposure and specific outcome measures, as well as a representative population in which to evaluate various asbestos exposure routes. In addition, a well-designed study of exposure status should adjust for SES and the health outcomes associated with slate roofs, including residence in a slate-dense area.

Despite these limitations, the observed asbestos exposure of ordinary citizens through various routes has provided new insights. Previously, industrialized areas containing many factories and poor urban areas may have housed working populations with greater exposure to asbestos, and residents in those areas might have had a lower SES, which has been associated with respiratory disease.

## 5. Conclusions

Substantial proportions of study subjects who had lived near asbestos factories, shipbuilding facilities, and slate roof-dense areas were exposed to asbestos through occupational, household, and slate roof routes. After controlling for sex, age, smoking status, and other asbestos exposure routes, we found that the occupational, household, neighborhood, and slate roof exposure routes were associated with LLF and PD; PD; LLF, PD, and LM; and LLF, PD, and LM, respectively. To our knowledge, this may be the first report of the health effects of slate roof exposure. However, this study is vulnerable to misclassifications of exposure and outcome measurements, information bias, and other potential confounders, including SES. A well-designed study is needed to overcome those limitations.

## Figures and Tables

**Table 1:** Prevalence and rates of radiological abnormalities by characteristics and exposure routes.

Variable	LLF			PD			LM			Subjects Total	
	Case	Rate	*p*-Value	Case	Rate	*p*-Value	Case	Rate	*p*-Value	No.	%
Sex			<0.0001			<0.0001			0.6063		
Female	120	1.9		211	3.4		61	1		6160	55.1
Male	264	5.3		295	5.9		45	0.9		5026	44.9
Age (years)			<0.0001			<0.0001			<0.0001		
≤29	0	0		9	1		1	0.1		943	8.4
30–39	6	0.2		25	0.9		7	0.3		2777	24.8
40–49	2	0.2		15	1.8		3	0.4		848	7.6
50–59	44	2.1		88	4.3		12	0.6		2064	18.5
60–69	132	5		165	6.2		34	1.3		2643	23.6
≥70	200	10.5		204	10.7		49	2.6		1911	17.1
Smoking status			<0.0001			<0.0001			0.7679		
Never	156	2.1		268	3.7		66	0.9		7302	65.3
Ex-smoker	140	6.6		154	7.2		21	1		2131	19.1
Present smoker	88	5		84	4.8		19	1.1		1753	15.7
Exposure routes			<0.0001			<0.0001			<0.0001		
No exposure	11	0.5		10	0.5		7	0.4		2022	18.1
Occupational	9	2.4		8	2.1		1	0.3		378	3.4
Household	1	0.4		1	0.4		0	0.0		274	2.4
Neighbor	204	3.4		286	4.8		50	0.8		5960	53.3
Slate	30	5.0		42	7.0		25	4.2		599	5.4
Occupational + Household	2	3.1		0	0.0		0	0.0		65	0.6
Occupational + Neighbor	69	12.5		58	10.5		6	1.1		553	4.9
Occupational + Slate	4	7.8		6	11.8		2	3.9		51	0.5
Household + Neighbor	12	2.1		18	3.1		1	0.2		580	5.2
Household + Slate	2	4.0		4	8.0		0	0.0		50	0.4
Neighbor + Slate	19	4.6		47	11.5		10	2.4		410	3.7
Occupational + Household + Neighbor	10	7.9		8	6.4		3	2.4		126	1.1
Occupational + Household + Slate	0	0.0		3	25.0		0	0.0		12	0.1
Occupational + Neighbor + Slate	7	13.0		7	13.0		1	1.9		54	0.5
Household + Neighbor + Slate	1	2.9		3	8.8		0	0.0		34	0.3
Occupational + Household + Neighbor + Slate	3	16.7		5	27.8		0	0.0		18	0.2
Total	384	3.4		506	4.5		106	0.9		11,186	100.0 *

LLF: lower lung fibrosis, PD: pleural disease, LM: lung mass, * total percent.

**Table 2:** Prevalence and rates of radiological abnormalities among cases of occupational and household exposure by job type and exposure duration.

Variable		Occupational Exposure *n* (%)			Household Exposure *n* (%)		
		LLF	PD	LM	LLF	PD	LM
Occupation type	No exposure	280 (2.8)	411 (4.1)	93 (0.9)	353 (3.5)	464 (4.6)	102 (1.0)
Production	Mine	6 (21.4)	5 (17.9)	0 (0.0)	0 (0.0)	1 (4.4)	0 (0.0)
	Cement	4 (14.3)	1 (3.6)	1 (3.6)	1 (4.6)	1 (4.6)	0 (0.0)
	Friction, joint sheet	2 (6.9)	5 (17.2)	0 (0.0)	0 (0)	2 (8.3)	0 (0.0)
	Textile	5 (11.9)	5 (11.9)	2 (4.8)	3 (14.3)	5 (23.8)	1 (4.8)
Use	Construction	23 (9.9)	17 (7.3)	2 (0.9)	2 (1.3)	2 (1.3)	0 (0.0)
	Chemical, power plant	4 (5.6)	5 (6.9)	0 (0.0)	5 (4.0)	9 (7.2)	1 (0.8)
	Plumbing, insulation	7 (5.6)	11 (8.7)	1 (0.8)	0 (0.0)	8 (6.0)	0 (0.0)
	Automobile, train repair	2 (5.7)	3 (8.6)	0 (0.0)	1 (2.9)	2 (5.7)	1 (2.9)
	Shipbuilding, ship repair	37 (9.1)	27 (6.6)	5 (1.2)	8 (2.1)	7 (2.0)	1 (0.3)
	Others	14 (5.5)	16 (6.3)	2 (0.8)	11 (4.5)	5 (2.1)	0 (0.0)
	*p*-value (Fisher’s exact)	<0.0001	<0.0001	0.3889	<0.0001	<0.0001	0.236
Duration	No exposure	280 (2.8)	411 (4.1)	93 (0.9)	353 (3.5)	464 (4.6)	102 (1.0)
	≤4 years	25 (5.7)	24 (5.4)	3 (0.7)	16 (2.4)	22 (3.3)	2 (0.3)
	5–9 years	15 (8.2)	18 (9.9)	3 (1.7)	1 (1.0)	4 (4.1)	1 (1.0)
	10–19 years	13 (5.7)	14 (6.1)	2 (0.9)	7 (5.3)	6 (4.5)	0 (0.0)
	≥20 years	51 (12.6)	39 (9.7)	5 (1.2)	7 (2.8)	10 (4.0)	1 (0.4)
	*p*-value (X^2^ test)	<0.0001	<0.0001	0.7942	0.2052	0.5580	0.2244

LLF: lower lung fibrosis, PD: pleural disease, LM: lung mass

**Table 3:** Prevalence and rates of radiological abnormalities among cases of neighborhood, slate roof, and slate roof-dense area exposure and exposure durations.

Variable		Neighborhood Exposure			Slate Roof and Slate Roof-Dense Area		
		LLF	PD	LM	LLF	PD	LM
Exposure type	No exposure	59 (1.7)	74 (2.1)	35 (1.0)	318 (3.2)	389 (3.9)	68 (0.7)
	Category1 *	302 (4.0)	390 (5.2)	68 (0.9)	35 (3.8)	75 (8.1)	8 (0.9)
	Category2 **	23 (10.5)	42 (19.1)	3 (1.4)	31 (10.4)	42 (14.0)	30 (10.0)
	*p*-value	<0.0001	<0.0001	0.6994	<0.0001	<0.0001	<0.0001
Duration	No exposure	59 (1.7)	74 (2.1)	35 (1.0)	318 (3.2)	389 (3.9)	68 (0.7)
	<4 years	30 (1.3)	55 (2.5)	14 (0.6)	3 (2.2)	11 (8.0)	1 (0.7)
	5–9 years	41 (1.9)	80 (3.7)	5 (0.2)	8 (5.4)	12 (8.1)	3 (2.0)
	10–19 years	157 (6.5)	194 (8.0)	30 (1.2)	10 (5.0)	17 (8.5)	3 (1.5)
	≥20 years	97 (10.6)	103 (11.2)	22 (2.4)	45 (6.0)	77 (10.4)	31 (4.2)
	*p*-value	<0.0001	<0.0001	<0.0001	0.0003	<0.0001	<0.0001

LLF: lower lung fibrosis, PD: pleural disease, LM: lung mass. * Factory (Neighborhood exposure), Slate roof (Slate roof and slate roof-dense area). ** Mine (Neighborhood exposure), Slate roof in slate roof dense area (Slate roof and slate roof-dense area).

**Table 4:** Trend tests of combined exposure source and duration variables and chest radiographic abnormalities.

Type	Type and Duration (Year)	LLF *n* (%)		PD *n* (%)		LM *n* (%)	
		No	Yes	No	Yes	No	Yes
Occupational	No exposure	9649 (97.2)	280 (2.8)	9518 (95.9)	411 (4.1)	9836 (99.1)	93 (0.9)
	Use & ≤19 years	698 (94.7)	39 (5.3)	694 (94.2)	43 (5.8)	732 (99.3)	5 (0.7)
	Use & ≥20 years	345 (87.8)	48 (12.2)	357 (90.8)	36 (9.2)	388 (98.7)	5 (1.3)
	Production & ≤19 years	102 (87.9)	14 (12.1)	103 (88.8)	13 (11.2)	113 (97.4)	3 (2.6)
	Production & ≥20 years	8 (72.7)	3 (27.3)	8 (72.7)	3 (27.3)	11 (100.0)	0 (0.0)
*p*-value	X^2^	<0.0001		<0.0001		0.0013	
	Trend test	<0.0001		<0.0001		0.1428	
Household	No exposure	9674 (96.5)	353 (3.5)	9563 (95.4)	464 (4.6)	9925 (99.0)	102 (1.0)
	Use & ≤4 years	598 (97.7)	14 (2.3)	596 (97.4)	16 (2.6)	611 (99.8)	1 (0.2)
	Use & ≥5 years	444 (97.2)	13 (2.8)	440 (96.3)	17 (3.7)	455 (99.6)	2 (0.4)
	Production & ≤4 years	62 (96.9)	2 (3.1)	58 (90.6)	6 (9.4)	63 (98.4)	1 (1.6)
	Production & ≥5 years	249 (92.3)	2 (7.7)	23 (88.5)	3 (11.5)	26 (100.0)	0 (0.0)
*p*-value	X^2^	0.3341		0.0137		0.1778	
	Trend test	0.2009		0.4177		0.0876	
Neighbor	No exposure	3392 (98.3)	59 (1.7)	3377 (97.9)	74 (2.1)	3416 (99.0)	35 (1.0)
	Factory & ≤19 years	6399 (96.9)	205 (3.1)	6314 (95.6)	290 (4.4)	6558 (99.3)	46 (0.7)
	Factory & ≥20 years	814 (89.4)	97 (10.7)	811 (89)	100 (11.0)	889 (97.6)	22 (2.4)
	Mine & ≤19 years	192 (89.3)	23 (10.7)	176 (81.9)	39 (18.1)	212 (98.6)	3 (1.4)
	Mine & ≥20 years	5 (100.0)	0 (0.0)	2 (40.0)	3 (60.0)	5 (100.0)	0 (0.0)
*p*-value	X^2^	<0.0001		<0.0001		<0.0001	
	Trend test	<0.0001		<0.0001		0.7600	
Slate roof	No exposure	9640 (96.8)	318 (3.2)	9569 (96.1)	389 (3.9)	9890 (99.3)	68 (0.7)
	Roof & ≤19 years	427 (96.6)	15 (3.4)	408 (92.3)	34 (7.7)	438 (99.1)	4 (0.9)
	Roof & ≥20 years	467 (95.9)	20 (4.1)	446 (91.6)	41 (8.4)	483 (99.2)	4 (0.8)
	Roof in dense area & ≤19 years	39 (86.7)	6 (13.3)	39 (86.7)	6 (13.3)	42 (93.3)	3 (6.7)
	Roof in dense area & ≥20 years	229 (90.2)	25 (9.8)	218 (85.8)	36 (14.2)	227 (89.4)	27 (10.6)
*p*-value	X^2^	<0.0001		<0.0001		<0.0001	
	Trend test	<0.0001		<0.0001		<0.0001	

LLF: lower lung fibrosis, PD: pleural disease, LM: lung mass.

**Table 5:** Logistic regression of radiologic abnormalities by exposure route.

Variable	LLF									PD									LM								
	Simple			Multiple *			Full **			Simple			Multiple			Full			Simple			Multiple			Full		
	OR	95%CI		OR	95%CI		OR	95%CI		OR	95%CI		OR	95%CI		OR	95%CI		OR	95%CI		OR	95%CI		OR	95%CI	
Occupational																											
No exposure	1.0			1.0			1.0			1.0			1.0			1.0			1.0			1.0			1.0		
Use & ≤19 years	1.9	1.4	2.7	1.1	0.8	1.6	1.2	0.9	1.8	1.4	1.0	2.0	1.0	0.7	1.4	1.2	0.8	1.6	0.7	0.3	1.8	0.6	0.2	1.4	0.7	0.3	1.7
Use & ≥20 years	4.8	3.5	6.6	1.9	1.3	2.7	2.3	1.6	3.3	2.3	1.6	3.3	1.2	0.8	1.7	1.6	1.1	2.3	1.4	0.6	3.4	0.9	0.4	2.3	1.3	0.5	3.4
Production	5.3	3.2	9.0	2.8	1.6	4.9	3.1	1.7	5.6	3.3	2.0	5.7	2.1	1.2	3.6	1.8	1.0	3.2	2.6	0.8	8.2	1.8	0.6	5.9	2.1	0.6	7.6
Household																											
No exposure	1.0			1.0			1.0			1.0			1.0			1.0			1.0			1.0			1.0		
Use & ≤4 years	0.6	0.4	1.1	1.2	0.7	2.0	1.1	0.6	1.9	0.6	0.3	0.9	0.8	0.5	1.3	0.7	0.4	1.3	0.2	0.0	1.1	0.2	0.0	1.5	0.2	0.0	1.7
Use & ≥5 years	0.8	0.5	1.4	1.1	0.6	2.0	1.3	0.7	2.4	0.8	0.5	1.3	1.0	0.6	1.6	1.2	0.7	1.9	0.4	0.1	1.7	0.4	0.1	1.7	0.6	0.1	2.4
Production	1.3	0.5	3.5	1.3	0.4	3.6	1.0	0.3	3.0	2.3	1.1	4.6	2.3	1.1	4.7	1.9	0.9	4.2	1.1	0.2	7.9	1.0	0.1	7.1	0.7	0.1	5.6
Neighbor																											
No exposure	1.0			1.0			1.0			1.0			1.0			1.0			1.0			1.0			1.0		
Factory & ≤19 years	1.8	1.4	2.5	2.7	2.0	3.6	4.1	2.8	5.8	2.1	1.6	2.7	2.7	2.1	3.5	4.8	3.4	6.7	0.7	0.4	1.1	1.0	0.6	1.5	2.7	1.4	5.2
Factory & ≥20 years	6.9	4.9	9.6	5.1	3.6	7.2	8.3	5.5	12.3	5.6	4.1	7.7	4.4	3.2	6.0	8.0	5.5	11.6	2.4	1.4	4.1	1.9	1.1	3.2	5.6	2.8	11.4
Mine	6.7	4.1	11.1	2.9	1.7	4.8	4.8	2.7	8.5	10.8	7.2	16.2	5.6	3.7	8.6	9.0	5.6	14.4	1.3	0.4	4.4	0.7	0.2	2.3	2.2	0.6	8.1
Slate roof																											
No exposure	1.0			1.0			1.0			1.0			1.0			1.0			1.0			1.0			1.0		
Roof & ≤19 years	1.1	0.6	1.8	0.8	0.4	1.3	0.7	0.4	1.3	2.1	1.4	3.0	1.7	1.2	2.5	1.6	1.1	2.3	1.3	0.5	3.7	1.1	0.4	3.0	1.1	0.4	3.0
Roof & ≥20 years	1.3	0.8	2.1	0.5	0.3	0.9	0.8	0.5	1.3	2.3	1.6	3.2	1.2	0.9	1.8	1.8	1.2	2.6	1.2	0.4	3.3	0.6	0.2	1.8	0.9	0.3	2.7
Roof in dense area	3.5	2.4	5.2	1.6	1.1	2.4	5.5	3.3	9.0	4.0	2.9	5.7	2.4	1.7	3.4	8.8	5.6	13.8	16.2	10.4	25.3	8.7	5.4	14.2	20.5	10.4	40.4

LLF: lower lung fibrosis, PD: pleural disease, LM: lung mass. * Adjusted for sex, age, smoking. ** Adjusted for sex, age, smoking + other exposure route.
